# An ANIr-based methodology to determine if two sequence-discrete populations are identical and identify cosmopolitan prokaryotic populations

**DOI:** 10.1093/ismeco/ycag068

**Published:** 2026-03-21

**Authors:** Roth E Conrad, Luis M Rodriguez-R, Blake G Lindner, Kenji Gerhardt, Konstantinos T Konstantinidis

**Affiliations:** Ocean Science & Engineering, School of Biological Sciences, Georgia Institute of Technology, Atlanta, GA 30332, United States; Department of Chemistry and Biosciences, Aalborg University, Aalborg 9220, Denmark; School of Civil & Environmental Engineering, Georgia Institute of Technology, Atlanta, GA 30332, United States; Center for Bioinformatics and Computational Genomics, School of Biological Sciences, Georgia Institute of Technology, Atlanta, GA 30332, United States; Ocean Science & Engineering, School of Biological Sciences, Georgia Institute of Technology, Atlanta, GA 30332, United States; School of Civil & Environmental Engineering, Georgia Institute of Technology, Atlanta, GA 30332, United States; Center for Bioinformatics and Computational Genomics, School of Biological Sciences, Georgia Institute of Technology, Atlanta, GA 30332, United States

**Keywords:** average nucleotide identity, biogeography, read recruitment, speciation, metagenome, intraspecies, differentiation

## Abstract

Although sequence-discrete species appear to dominate microbial communities, readily distinguishing between distinct populations of a species recovered from different short-read metagenomic samples is challenging due to technical limitations associated with read length. To close this gap, we developed a novel algorithm to evaluate which reads in a metagenome belong to a target population based on the distribution of sequence identities of reads aligned to a reference sequence, which are filtered using a Kernel density estimation (KDE) as a flexible alternative to the commonly used static 95% nucleotide identity cutoff. Subsequently, we employed the average nucleotide identity of reads (ANIr) aligning above the KDE threshold, and resampling techniques for estimating the confidence intervals of ANIr values, to quantify intrapopulation sequence diversity and compare populations across globally representative marine samples. Most populations showed high ANIr in only a few samples at similar depths and decreased ANIr and increased gene-content difference between samples where a closely related population is detected (e.g. same 95% ANI-based species). Accordingly, ANIr correlated with the physical distance between the samples, and only a few truly cosmopolitan populations were identified. Among the latter, *Alteromonas macleodii* [97% average amino-acid identity (AAI) to the type genome] and *Prochlorococcus marinus* (79% AAI) showed high relative abundance in both surface (0–200 m) and deep (>1000 m) samples. These results suggest that microbial communities under different environmental conditions share very few identical and abundant populations and provide a highly needed methodology to track such populations over space and time, in marine or other habitats.

## Introduction

Recruitment of short-read sequences to isolate, metagenome-assembled (MAGs), or single-cell amplified (SAGs) genomes is commonly used in metagenomic surveys to detect species and ascertain their relative abundance in a sample. The first studies to pioneer these methods relied on the *blastn* algorithm and visualization of tabular Basic Local Alignment Search Tool (BLAST) output tables via recruitment plots [1–3]. Datasets have grown exponentially in the past decade, necessitating methods capable of leveraging newer and faster read mappers. Yet, despite advancements in read mapping software, results are often automatically summarized into standard coverage metrics (e.g. sequencing breadth, sequencing depth, and relative abundance) based on static alignment quality thresholds for mapped reads, such as 95% nucleotide identity [4–6]. This type of automation is convenient but can also lead to confounding results from mapped reads, which represent related but distinct genotypes or species co-occurring in the sample [7–9].

Recent work has shown that robust detection of a reference target and estimation of its relative abundance is typically achieved if the sequencing breadth provided by mapped reads covers at least 5%–6% of the reference sequence, with 10% proposed as a conservative, general-purpose threshold [10]. However, since metagenomic populations are not well defined, and since target vs. nontarget population level filtering of the mapped reads is not part of standard workflows, the standard method to compute coverage metrics may produce inaccurate or false positive results. Such results could emerge when co-occurring closely related (but distinct from the target) species are present or as populations in one or more subsequent metagenomic samples diverge from the reference sequence used for read mapping. This issue also applies to strain-resolved methods with tools that leverage short-read mapping results to make inferences based on the single-nucleotide variants (SNVs) identified [8, 11, 12].

**Figure 1:**
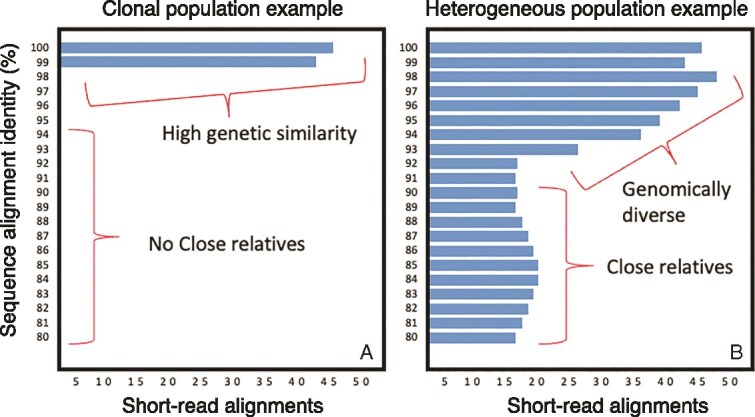
A hypothetical example of short-read sequence identity distributions for a clonal vs. a heterogeneous species. The sequence-identities of short-reads mapped to a reference genome sequence (y-axis) are plotted against the number of short-read alignments (x-axis) per each 1% bin of the y-axis. The resulting histograms represent (A) a clonal population and (B) a heterogeneous population with one or more co-occurring close relatives.

Microbial species exhibit varying levels of sequence diversity resulting in some species harboring greater intraspecies diversity than others (or, conversely, being more clonal) (Fig. 1) [9, 13–17]. Indeed, natural populations of microbial species may exist as combinations of multiple strains, genomovars, and phylogroups, with genomovars defined as collections of genomes showing typically >99.5% ANI and genomes of the same strain showing >99.99% ANI [13, 14, 18]. We refer to these co-existing combinations of intraspecies units as subpopulations as they are distinct components constituting the local representatives of the total species population, and typically representing the read sequence captured in a metagenome sample matching to a target species. Metagenomic signal from clonal populations lacking co-occurring subpopulations or close species-level relatives is easy to detect and quantify based on standard analysis approaches, as read-mapping results are highly reliable and typically free of false positives resulting from partial sequence similarity (Fig. 1A). However, challenging scenarios could emerge where two related clonal populations or species (e.g. sharing 92%–95% ANI) co-exist and the reads from one population are recruited by another (Fig. 1B) or in cases of nonclonal (diverse) populations. The former scenario is especially challenging when a representative genome for only one of these species is available, preventing competitive read mapping against representative genomes of both species. Even in cases that representative genomes are available, however, it is possible that many reads could achieve identically scored alignments to either genome, making it impossible to decide from which genome (species) such reads originated (and one of the two species could be absent or much less abundant than the other).

In this study, three new analytical and algorithmic approaches were developed to drive high-throughput, robust species and subpopulation level analyses using short-read metagenomic data. We showcase the usefulness of these algorithms by applying them to available oceanic metagenomes to identify cosmopolitan populations; that is, populations that are identical across depths (i.e. vertically within the water column, and stratified water masses) and ocean basins (i.e. horizontally around the globe). We focus our work and approach validation to the ocean environment because of its relevance for human activity but also that the vertical and horizontal distribution of microbial populations is somewhat understood [19–22], which allowed us to have expectations about the results to be obtained. The results obtained and approaches developed should be directly applicable to metagenomes from other habitats.

## Materials and methods

### Study data, approach, and reasoning

**Figure 2:**
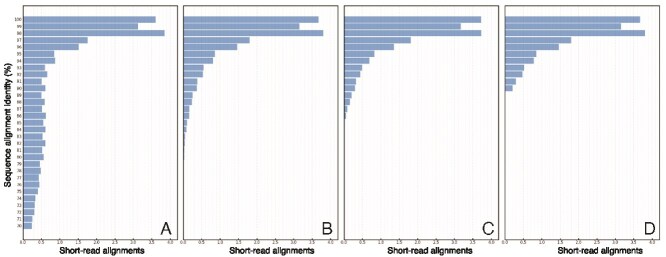
Differences in short-read sequence identity distributions between mapping algorithms for the exact same dataset. The sequence identity of short-read alignments to the same reference sequence (y-axis) is shown for (A) *blastn*, (B) *megablast*, (C) MagicBlast, and (D) Bowtie2 algorithms as histograms with the slower to the faster algorithms arranged from left to right. Notice that faster algorithms report results down to ~90% sequence identity, i.e. they tend to map only highly identical reads to the reference in order to be faster. As mentioned in the text, read mapping patterns can vary depending on species and sample. This data is intended to illustrate the effect of just the mapping algorithm on the patterns.

Short-read metagenome samples collected from the Gulf of Mexico (GoM) Station 5 and other studies of the North and South Atlantic and the Pacific Oceans ([23] and Sup. File 1) were selected for this analysis. Metagenomic shotgun short reads from these datasets were mapped to the 95% ANI-based cluster representative MAGs (rMAGs), recovered previously from the GoM [23]. Read mapping was performed using the *blastn* algorithm from BLAST+ [2], which is more sensitive in finding moderately related reads to the reference sequence according to the analysis shown in Fig. 2. To gain intuition about read mapping results from natural marine populations, develop methodology, and identify candidate populations that span several depths or ocean basins, several Python packages were leveraged to construct novel data processing capabilities that incorporated flexible user parameters as described below.

### Selecting reads representing the target population using the Kernel density estimates

One approach to identify co-occurring distinct species (or subpopulations) present in a metagenome is to use read recruitment plots to estimate by eye (manually) an appropriate filtering threshold in the distribution of sequence identities (abbreviated as seqIDs hereafter) of mapped metagenomic short-reads that reflects such species. The key underlying assumption, which is adopted by this study, is that a bi- or multimodal distribution (two or more peaks in the density curve) indicates the presence of two or more closely related (but distinct), co-occurring species or subpopulations relative to the reference genome used. However, this task is time-consuming and often overlooked (e.g. replaced by a static 95% seqID cutoff). In addition, seqID distributions commonly exhibit nonparametric distributions, and thus different researchers may not agree on the best cutoff to choose [4, 24]. Kernel density estimation (KDE) is a nonparametric method that utilizes kernel smoothing to estimate the probability density function of a random variable [25, 26]. KDE can be applied to empirical data measurements such as the distribution of seqIDs to assess the type of distribution and identify patterns in distributions.

**Figure 3:**
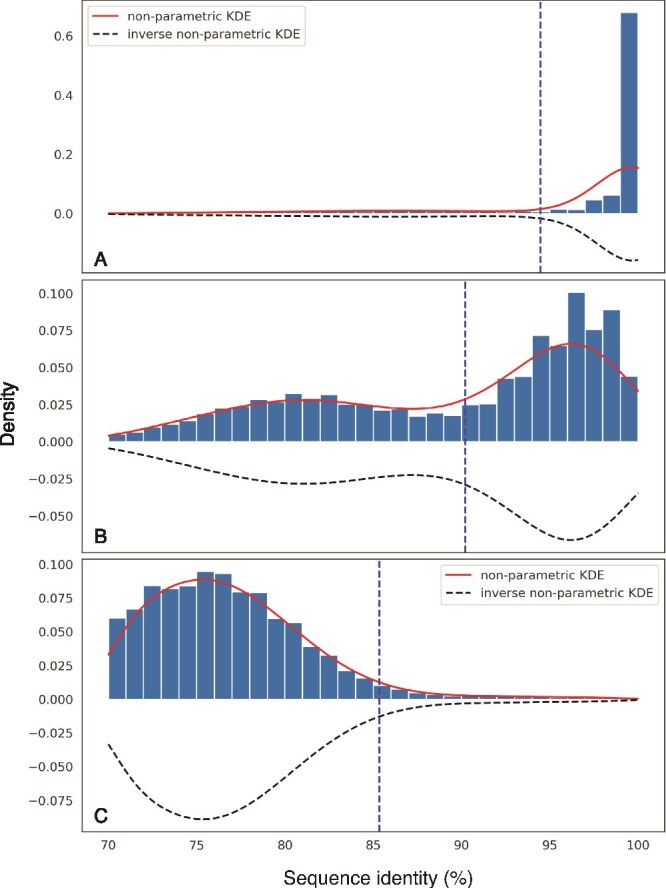
Examples of KDE fitting to identify the distribution of nucleotide identities of mapped reads from different metagenomes against the same reference genome. The sequence identity of short-read metagenomic alignments with the best match to the reference sequence (rMAG) is recorded for each read (x-axis) and the normalized counts (density) are computed (y-axis) for each reference sequence separately. The kernel density estimate (solid curve) and its inverse (dotted dashed curve) are subsequently computed. Three examples are shown where the metagenome population is: (A) highly similar to, (B) somewhat similar to, and (C) not similar to the reference sequence. The vertical dashed line indicates the local minimum identified by the peak finding algorithm with an adjustment applied to provide a more conservative estimate as explained in the text. Note the bimodal shape of the distribution in B indicating one or more co-existing close-relative populations (in the range of 75%–85% nucleotide sequence identity) with fewer reads at high sequence identity (in the range of 97%–100% nucleotide sequence identity) compared to A. Data shown in this figure is from our Station 5 GoM samples at 1470 m (A), 300 m (B), and 88 m (C) mapped to the selected archaeal GoM rMAG. The rMAG used was classified as a novel member (phylum-level) of the archaeal domain by MiGA, with the closest RefSeq match being *Aciduliprofundum boonei* T469 (39.05% AAI). See text and Fig. 5 for additional details.

To advance this task, the *scipy.signal.find_peaks()* function, which identifies the local maxima in a distribution, was applied to the inverse of the seqID KDE, since using the inverse will identify the local minima. Specifically, we wrote an algorithm to parse tabular *blastn* or SAM output from common read mapping tools, collect the seqIDs, fit the KDE using the *scipy.stats.gaussian_kde()* function, and identify the peaks of the inverse KDE. A few selected examples taken from our GoM data are shown in Fig. 3. KDE functions have a bandwidth parameter that affects the amount of smoothing applied by the kernel. This bandwidth value is adjustable by the user with an optional parameter controlling KDE smoothing so it may be adjusted depending on the seqID pattern of read recruitment for the target population of interest or to match the scale of the seqID distribution dependent on the range of the data. We set the default bandwidth value to 0.25, which we found to work well in general for *blastn* results with a range of 70%–100% sequence identity (the typical range observed in recruitment plots). Additionally, due to the KDE smoothing, the local minima identified from the KDE curve frequently appeared to lag compared to the data represented as a histogram. Thus, a valley modifier (valley referring to minima) of +3 is used as the default in the code to adjust the local minimum higher, and thus produce a more conservative selection (reduced reads identified as false positives with respect to representing the target population). The valley modifier is also a user-adjustable parameter to allow flexibility in making more or less conservative selections and to adjust for the range of the data observed.

### Estimating sequence coverage metrics for the target population

**Figure 4:**
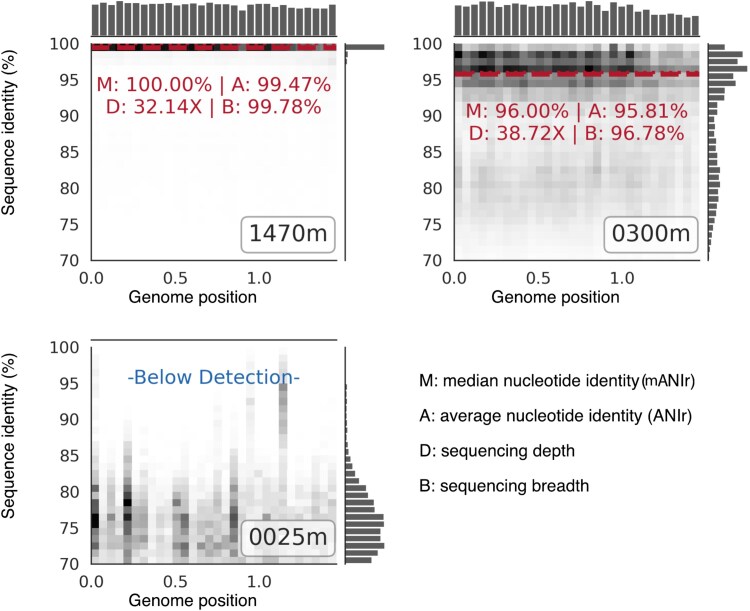
Examples of the output of the RecPlot minis tool. RecPlot minis output shows the genome position of read alignments along the x-axis and the sequence identity of aligned reads along the y-axis. The top and right marginal plots display quantitative histograms of the read distributions for all mapped reads along their respective axes (the right marginal plot is similar to those shown in Fig. 1 and Fig. 3), and a density-based grid in the main plot displays the proportional quantity of reads for each given cell in the grid. Black indicates a high density of reads in a cell, and white indicates no reads in a cell. If the sequencing breadth from the target population’s reads is ≥10%, the population is robustly detected based on our previous work [10], and a red dashed line is drawn indicating the ANIr of the target population to the reference sequence along with explicit values for (M) mANIr, (A) ANIr, (D) sequencing depth, and (B) sequencing breadth. Otherwise, no such metrics are calculated because the target population is below the limit of robust detection and quantification of the metagenomic effort [55, 56]. These particular RecPlot minis have the depth in meters amended in the bottom right corner corresponding to the sample depth of the metagenome. Panel A shows a population that appears to be highly clonal to the reference sequence. Panel B shows a reference population that is detected and its metagenomic population shows substantial intraspecies diversity (lower ANIr), either because the reference genome is slightly divergent compared to the population (and thus is not a 100% match to many sequencing reads), or the population is indeed diverse (if the MAG is a good representative of the population, which is typically the case when the MAG originates from the same metagenome). Note also the presence of one or more co-occurring relative species with lower abundance evidenced by the lower peak around 80% sequence identity in Panel B. Panel C shows that the target population is below detection but that one or more members of the community (possibly belonging to the same genus) are present and share a few hotspots of conserved sequence (which represent false-positive signal for the detection of the target population). The same data as in Fig. 3 are used, except for the shallowest depth metagenome (25 vs. 88 m for Figs 4 vs. 3, respectively).

**Figure 5:**
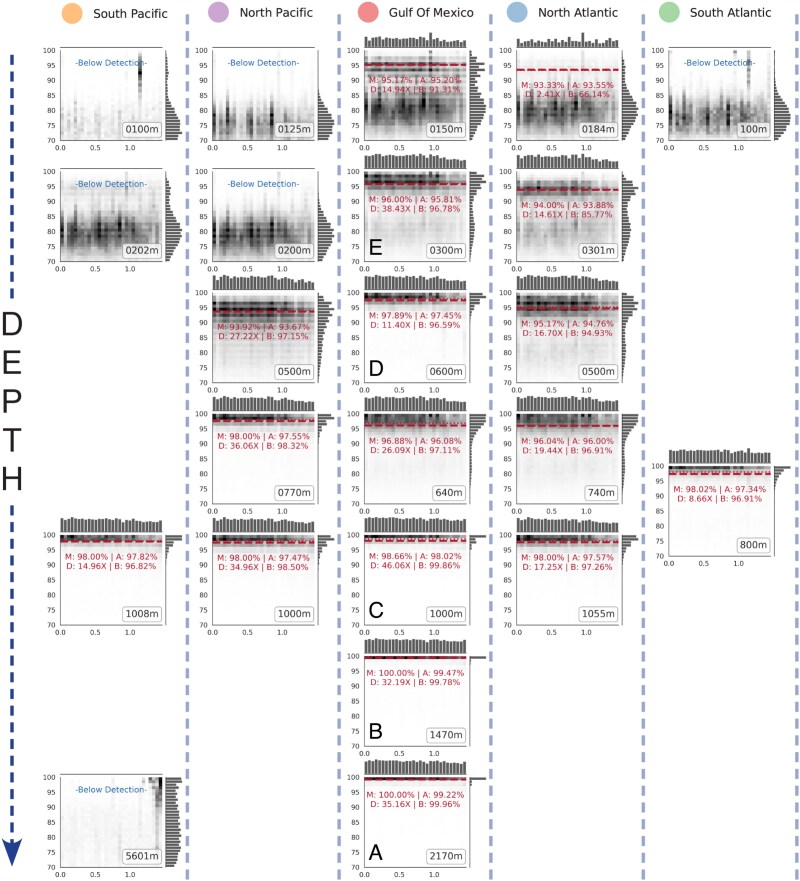
Arranging RecPlot minis to visualize results across several samples simultaneously. RecPlot minis are arranged according to ocean basin and ocean depth. This allows the researcher to quickly identify where a population is detected or not, and how the reference population, represented by the (same) reference genome used in the plots, changes or not across samples. Note that for this analysis, the same reference sequence (rMAG) was used for read recruitment in all samples, and the rMAG used in this example originated from the GoM sample EN59 collected from 2170 m at Station 5 shown in Panel A. The rMAG used here was classified as a novel member (phylum-level) of the archaeal domain by MiGA. The closest RefSeq match to this rMAG shared 39.05% AAI and was a genome for *Aciduliprofundum boonei* T469, which is a thermoacidophilic archaeal assigned to the “Deep-Sea Hydrothermal Vent *Euryarchaeota* 2 (DHVE2)” clade. This clade is widely found as part of deep-sea hydrothermal microbial communities [46, 47]. The results from Panels A–E are referenced in the text and in Figs 7 and 8.

The local minimum with maximum identity (i.e. the valley closest to 100% sequence identity) with the applied valley modifier was used to set the seqID threshold for the reads representing the target population. That is, reads with a sequence alignment identity greater than or equal to this threshold were retained as matches to the target population, and reads below this threshold were discarded as distant matches from a related but nontarget population (Figs 1 and 2). Sequencing depth and breadth along with the median and average nucleotide identity of the reads (mANIr and ANIr) aligned to the reference sequence were computed using the target population reads for each of the 209 rMAGs and the 39 GoM samples used in this study (see Fig. 4 for examples). That is, each reference sequence is held constant against the read alignments from each sample. We developed a new plot that facilitates the review of many results on the screen simultaneously, which we called RecPlot minis, inspired by the RecPlot tool previously developed by our team (Fig. 4) [4, 24]. RecPlot minis can be arranged positionally to match a sampling scheme (such as ocean basin and depth) and show the results from many metagenomes against a single reference sequence on a single page (Fig. 5). The mANIr is reported in addition to the ANIr because the sequence identity distributions from short-read alignments frequently exhibit non-normal shapes having long tails to the left or clustering tightly to the right against the 100% boundary; hence, the two statistics may sometimes differ from each other, and mANIr may reflect the long tails better. Such long tails are due, at least in part, to the mere effect of the length of short reads. For example, one base pair difference in 1000 bp long read translates to 0.1% difference (or 99.9% identity) whereas for a 100 bp read translates to 1% difference. Therefore, a small number of reads that may map with lower identities compared to the dominant identity pattern could represent such spurious results caused by the clustering of a few single-nucleotide polymorphisms (SNPs) in the genome and the short-read length, rather than closely related (to the reference genome), distinct, co-occurring species. In these cases, the median likely provides a better estimate for the expected value, and it is also informative to know the difference between mANIr and ANIr. Note also that we suggest preserving the term ANI for whole-genome comparisons of isolate genomes or MAGs/SAGs, either based on genes or 1 Kbp-long consecutive fragments, which includes intergenic regions, and to use ANIr/mANIr for read-based assessment of ANI.

### Estimating normalized relative abundance of genes and differentially abundant genes

Protein coding sequences (CDS) were predicted using Prodigal version 2.6.3 [27] with default settings and annotated with MicrobeAnnotator v1.0.4 with default settings, Diamond v2.0.1 and Kofam, Swissprot, TrEMBL, and RefSeq databases created 10 February 2021 [28, 29]. The positional information from read alignments on contigs and CDS on contigs was used to calculate the sequencing depth for each gene (CDS). Gene sequencing depth was then normalized by the rMAG relative abundance to set all gene relative abundances in reference to the rMAG relative abundance in each sample. These normalized relative gene abundances were then used to compute per gene difference between pairwise sample comparisons. Pairwise differences between samples were stored in an array, and the *numpy.quantile()* function [30] was used to retrieve genes found with difference in the bottom 2.5% and top 97.5% of all gene differences for an individual rMAG between two samples. These genes represented those with a significant difference (*P* ≤ .05) compared to the average difference in relative gene abundance of an rMAG in two different samples. These differential gene abundance functions have been incorporated into the *08b_BlastPlus_CoverageMagic_Basic.py* and *08d_MagicBlast_Differential_Genes.py* scripts available from the GitHub repo associated with this manuscript.

## Results and discussion

### Distinguishing between target vs. nontarget sequences and the associated confounding factors

Most read mapping tools can report results down to ~90% sequence identity (SeqID; Fig. 2), unless they are designed for aligning divergent sequences (e.g. *blastn*; Fig. 2A). Thus, the SeqID distribution from short-read mapping results could be examined for a signal of single or multiple peaks or valleys in the density distribution of read mapping results that would represent distinct co-existing subpopulations. A valley between two peaks would indicate the presence of a similar but divergent subpopulation or a closely related species to the reference sequence that is represented by the second, lower identity peak. Likewise, a lack of read recruitment at 100% sequence identity and a lower peak (at 97% or below, for example) would indicate population divergence from the reference genome. These are important aspects that should be considered during standard metagenomics analyses. Some of these aspects such as automatic peak detection of coverage patterns across the genome have been implemented, for instance, in our read-recruitment tool of the enveomics collection [24] and the newer, updated version of it, the RecruitPlotEasy [4], but other aspects such as how to identify same (target) vs. nontarget population reads remain challenging to account for.

Related but distinct species or subpopulations can vary in two parameters. First, populations can exhibit allelic differences in core or shared genes, which is commonly investigated using SNV or SNP analyses, or with population diversity metrics such as π (pi) [31–33]. Similarly, the average nucleotide identity of the reads (ANIr) alinged to the reference sequence can be used to measure how similar or not a population in a metagenomic sample is to the reference sequence [12, 34]. For instance, for a 150 bp read, a sequence similarity score of 98% equates to three SNVs; thus, a 98% ANIr indicates that the sampled population has three SNVs per 150 bps, on average, compared to the reference sequence. Accordingly, comparing ANIr between samples holds promise in revealing closely related yet differentiated populations, although a rigorous approach to determine when populations are differentiated significantly from each other has not yet emerged [34]. Second, species or subpopulations within species can exhibit gene content differences (presence vs. absence) in accessory or flexible genes as well as differences in the relative frequency of those genes between populations and subpopulations [14, 35]. Tools for differential gene abundance such as DeSeq2 have been applied to metagenomics studies, but the assumptions made by these tools are frequently violated by metagenomics surveys where biological replicates and control vs. experimental samples are not available [36]. The two parameters, SNVs and gene content, usually correlate with each other, i.e. gene content differences increase with lower core genome relatedness [37], although the relationship at the species level can vary depending on the organism considered [13]. To improve accuracy in the computation of ANIr or other population diversity metrics as well as in relative genome and gene abundance estimates, a new approach is needed to identify reads belonging to the population of interest (i.e. the target population) vs. reads belonging to nontarget populations (i.e. closely related and co-existing species or subpopulations) and assess intrapopulation structure [38–41].

### Detecting the “same” population across samples

**Figure 6:**
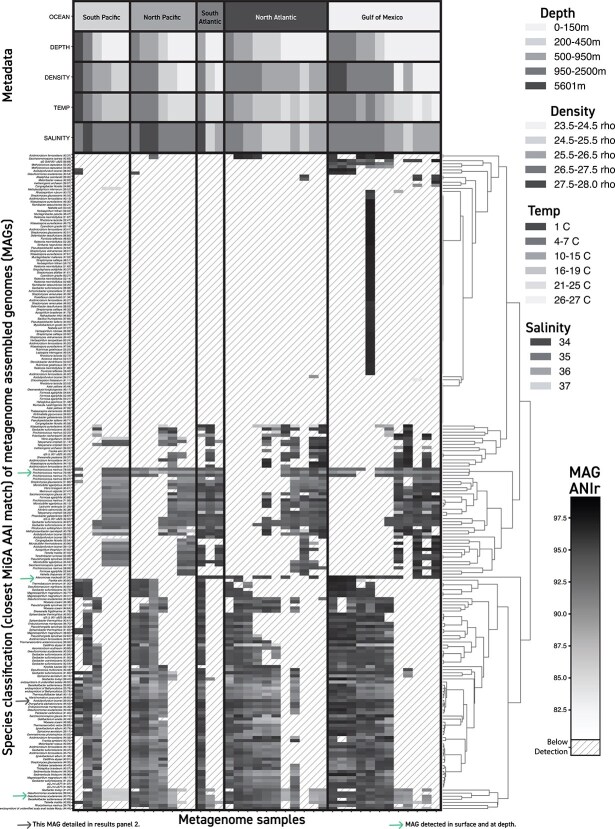
Target population ANIr of rMAGs against metagenomes from different ocean basins and depths. Each row of the heatmap displays ANIr data for short-read sequence alignments identified above the target population threshold for individual rMAGs against metagenome samples from different depths and ocean basins in each column. Columns are organized by depth from deeper on the left to surface on the right within each ocean basin. Rows are organized based on hierarchical clustering of the ANIr matrix. Black indicates high ≥97.5% ANIr of a metagenome population to the reference sequence, and white to light grey indicates ≤82.5% ANIr. Diagonal gray lines indicate that a population matching that reference was not detectable in that sample. The three rMAGs marked by green arrows were identified in surface and deep-water samples in at least one ocean basin. The black arrow marks the rMAG whose data is shown in Figs 5, 7, and 8.

Following our previous work [10], a target species was considered detectable in a given sample if sequencing breadth ≥10% was achieved from the target population reads (Figs 4 and 5), which overcomes spurious matches from highly conserved genes and nondiagnostic signal from recent horizontal gene transfer. [Note that estimating the sequencing breadth from the sequencing depth based on their established relationship [10] can sometimes be inaccurate, especially in cases where depth is high, but all reads map to only a few regions of the genome such as those encoding for the rRNA genes or recently horizontally transferred genes. Hence, we suggested to directly estimate breadth based on the unique (nonredundant) bases of the reference sequence covered by mapped reads]. Using this threshold and the reads identified to represent the target population based on our KDE-based approach described in the Methods to calculate the sequencing breadth, we first aimed to identify which of the previously reported 209 rMAG recovered from the GoM depth profile metagenomes [23] were present in selected GoM metagenomes and metagenomes from other ocean basins. We found that 74 of the 209 rMAGs (35.41%) were detectable in only a single sample (the sample that the MAG was recovered from), and 135 (65.59%) were detectable in at least two samples (Fig. 6). Of the rMAGs found across multiple samples, we qualitatively observed three major patterns (Fig. 6). First, there was a clear distinction between surface populations and deeper water populations in all ocean basins, which echoes the results of previous studies [20, 22]. Many of these populations exhibit some overlap in the intermediate (middle) depths, but interestingly, a few species were detectable in both the surface and in deep water samples showing similar ANIr, which has not been reported previously to the best of our knowledge. Second, similar—but not necessarily identical—populations were detected at similar depths across ocean basins. And third, the ANIr fluctuated between samples, but generally, the ANIr was more similar between ocean basins at similar depths, as well as at similar density and temperature, than between different depths (Fig. 6).

According to results from MiGA [42], the rMAGs that were detected across depths (deep and shallow) with similar ANIr shared closest RefSeq matches to *Alteromonas macleodii* [97% average amino-acid identity (AAI)], *Prochlorococcus marinus* (79% AAI), and *Desulfuromonas soudanensis* (40% AAI). The populations whose rMAG matched *A. macleodii* were not detected in the North or South Pacific but were detectable with ANIr ≥97% throughout the entire water column (surface down to 2170 m) in the North and South Atlantic as well as the GoM. The South Atlantic and North Atlantic populations shared around 97% and 98% ANIr to the rMAG reference sequence, respectively, and the GoM surface populations showed 98% ANIr while the deeper water populations showed 99% ANIr and above. The populations whose rMAG was similar to *P. marinus* were most prominent in the North Pacific, South Atlantic and GoM. They were not detectable in the North Atlantic or South Pacific samples below the photic zone except for the deepest South Pacific sample at 5601 m. The populations whose rMAG was most similar to *Desulfuromonas soudanensis* had higher ANIr in deeper waters and lower ANIr approaching the surface. They were more prominent in the surface waters of the North Atlantic and the GoM than in other ocean basins.

### Testing the significance of average nucleotide identity of reads differences with bootstrap and permutation methods: is the “same” population indeed the same?

To more precisely estimate any ANIr differences between populations from two different samples and whether or not they should be considered identical, including the populations mentioned above to share high ANIr values between surface and deep samples, bootstrap (repeated experiments by random sampling with replacement) and permutation test (re-randomization test or shuffle test) methods were developed and utilized to build probability distributions and test significance. To do this, a single rMAG was used as the example for the remainder of this analysis that had the highest quality score (92.3%; based on MiGA analysis) among the set of rMAGs with ≥5× sequencing depth and ≥50% sequencing breadth in five or more samples. This rMAG was taxonomically classified by MiGA as *Archaea* at the domain level (MiGA *P* = .000112462) with no significant classification beyond that level [42], suggesting that it likely represented at least a novel family of *Archaea*. The closest matches for this rMAG in the RefSeq, Delmont, and Tully databases [42–45] were 39.05%, 74.55%, and 78.28% AAI, respectively. The closest RefSeq match was *Aciduliprofundum boonei* T469 (39.05% AAI), which belongs to a thermoacidophilic archaeal clade known as the “Deep-Sea Hydrothermal Vent *Euryarchaeota* 2 (DHVE2)” and is widely found as part of deep-sea hydrothermal communities [46, 47]. The rMAG consisted of 251 contigs with an N50 of 10 739 bp, 59.23 G + C% content, 1441 predicted protein CDS, and a total length of 1 458 496 bp.

This rMAG was recovered from GoM Station 5 at Depth 9 (2170 m). A similar MAG was also recovered from Depth 8 (1470 m) and was included in the same 95% ANI-based species cluster with 99.4% ANI. This archaeal rMAG was detected at the species level (ANIr ≥95%) in 17 of our metagenome samples as shallow as 150 m down to 2170 m in the GoM water column and across all five ocean basins between 800 – 1055 m. (Fig. 5). We found that the population in the 2170 m GoM metagenome (where the rMAG was recovered) was highly homogeneous (or clonal, similar to the example in Fig. 1A) with an ANIr of 99.22% and an exact match ratio (EMR) of 0.73 (Fig. 5A). The EMR was calculated as the number of reads mapping ≥99% sequence alignment divided by the reads mapping below 99% but above the target population cutoff identified by the KDE analysis. Higher EMR values indicate a more clonal population and more related to the reference sequence. Moving further up the water column, we observed the sampled populations to become more heterogeneous (e.g. wider spread in read alignment sequence identities) with decreasing ANIr and EMR values when the reads from shallower depths and different ocean basins were mapped against the 2170 m reference sequence. The population at 800 m–1000 m, identified in all five ocean basins, became more heterogenous/divergent (relative to the rMAG from 2170 m depth) than in the two deep GoM samples (1470 m and 2170 m) with EMRs of 0.22–0.45 and ANIr of 98.02% in the 1000 m GoM sample and ~ 97.5% ANIr at similar depths in the North and South Atlantic and Pacific Oceans (Fig. 5A, B, and  the  entire  row  for  C). Moving further up the water column in the GoM and North Pacific and Atlantic, EMRs approached 0 and ANIr decreased to as low as 93.33% (i.e. below the common species boundary) until the population was no longer detected above 150 m. Note that a decrease in ANIr could occur due to an increase in microdiversity or divergence of the local population from the reference genome. Both scenarios as well as a combination of them are possible in this case, and one way to gain further insights into this issue is by using, for instance, an endemic MAG recovered from each sample to recruit reads and quantify the microdiversity based on the (local) MAG. This was outside the scope of our objective here, however, and thus we did not pursue this line of investigation further. Of note, sample population ANIr divergence from the reference rMAG was most strongly correlated with water density (Pearson *r* = 0.94, *P*<.00005, Sup. Fig. 1), although collinearity was also observed between density, depth, and temperature.

**Figure 7:**
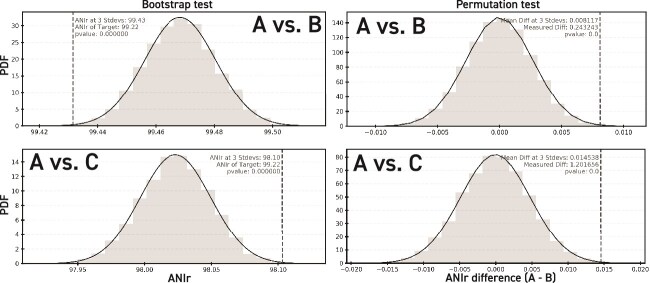
Bootstrap and permutation tests for the significance of ANIr differences between samples. Bootstrap and permutation test results for the archaeal population used in Fig. 5A, where its rMAG was searched against the metagenomes used in Fig. 5  B and C. The light gray histogram and black curve show the probability distribution of ANIr (bootstrap tests; left) or difference in ANIr between samples (permutation tests; right) generated by the corresponding random resampling approach. The vertical dashed line denotes the measured ANIr or ANIr difference between the samples, respectively. Note that in all instances, the measured value is beyond three standard deviations of the resampled probability distributions, and the *P*-value is <.05, revealing significant differences in the populations detected between the samples used in this example.

To better understand the significance of the changes in ANIr of (any) two target populations, detected using the same rMAG, from different samples (e.g. Sample A and Sample B), and since metagenomic surveys do not typically include biological replicates, or control and experimental group samples [39, 41], a bootstrapping approach [48] was used to build a probability distribution of expected ANIr for any given target population. To do this, a set of 10 000 resampling experiments were performed where each experiment consisted of randomly selecting, with replacement, a set of seqIDs (or reads) of the target population from one sample (Sample A) that was 2% of the size of the total seqIDs detected for the target population in that sample and recording the new ANIr. The result from 10 000 experiments is a bootstrapped probability distribution that was used to test the null hypothesis that the target population detected in Sample A was the same as the target population detected in Sample B by comparing the ANIr of Sample B to the bootstrapped probability distribution produced for Sample A and vice versa (Fig. 7, left panels). Note that for this analysis to make sense, the reference rMAG used for the target population should ideally be assembled from Sample A and be a good match to the reads in that sample. Then, this same rMAG from Sample A is held constant in read mapping and identification of the target population for Sample B and additional samples. Finally, if the ANIr from Sample B was outside three standard deviations from the mean ANIr, or in the bottom 2.5% or top 97.5% percentile in the bootstrapped ANIr probability distribution for the target population from Sample A, then the null hypothesis that the two populations were the same (i.e. had the same ANIr) was rejected. An additional note about the approach for this analysis is that using a nonendemic reference genome for Sample A fundamentally changes the interpretation of these results. Therefore, we advise that researchers perform these types of analyses using MAGs (or isolate genomes) recovered from Sample A to compare with Samples B, C, etc.

To further support this intuition about the significance of ANIr differences, a permutation test [49] was used to build a probability distribution of the expected difference in ANIr between two target populations, detected using the same rMAG, with reads recruited from different samples (i.e. Sample A and Sample B), assuming both target populations are sampled from the same, larger population. To do this, a new set of 10 000 resampling experiments was performed, where each experiment consisted of combining the target population seqIDs from Sample A and Sample B, randomly splitting the combined seqIDs into two new separate sets equal in size to the two seqID inputs, computing new ANIr for each random set, and recording the difference between the new ANIr values. The result from 10 000 repeated experiments is a probability distribution of the difference in ANIr between two samples taken from the same population. This probability distribution was used to test the null hypothesis that there is no significant difference in ANIr between two target populations, detected using the same rMAG, with reads recruited from different samples (Fig. 7, right panels). If the ANIr difference between the target populations from Sample A and Sample B fell outside three standard deviations or in the bottom 2.5% or top 97.5% percentile in the permutation test’s ANIr difference probability distribution, then the null hypothesis that the two populations were the same was rejected.

We found that even the ANIr difference of 0.24 found between our GoM samples at depths 1470 m and 2170 m was statistically significant in our permutation test. Likewise, based on the bootstrapping results, it was highly unlikely (*P* < 1.00E-6) to see the 99.22% ANIr from the 2170 m sample’s population (Figs 5A and 7, top right panel) based on the short-read sequence alignments detected from the 1470 m sample’s population (Figs 5B and 7, top left panel). These results showed that even relatively small changes in ANIr could indicate significant differences at the genomic level between what appear to be highly similar populations sampled from water masses of similar—but not necessarily identical—physicochemical properties such as density and temperature. These resampling tests cannot reveal what the exact differences are between the populations, but, like the commonly used ANOVA, they can indicate when a difference is significant, and thus worth investigating further, as necessary. We have also outlined an approach for how such investigations could be performed recently [13, 14]. The difference in ANIr could likely represent either genomic adaptation to the hydrostatic pressure difference between the depths in core or shared genes [19] and/or differences in abundance, including presence/absence, of two or more genomovars shared between the populations [13].

### Testing the significance of gene content differences between samples

**Figure 8:**
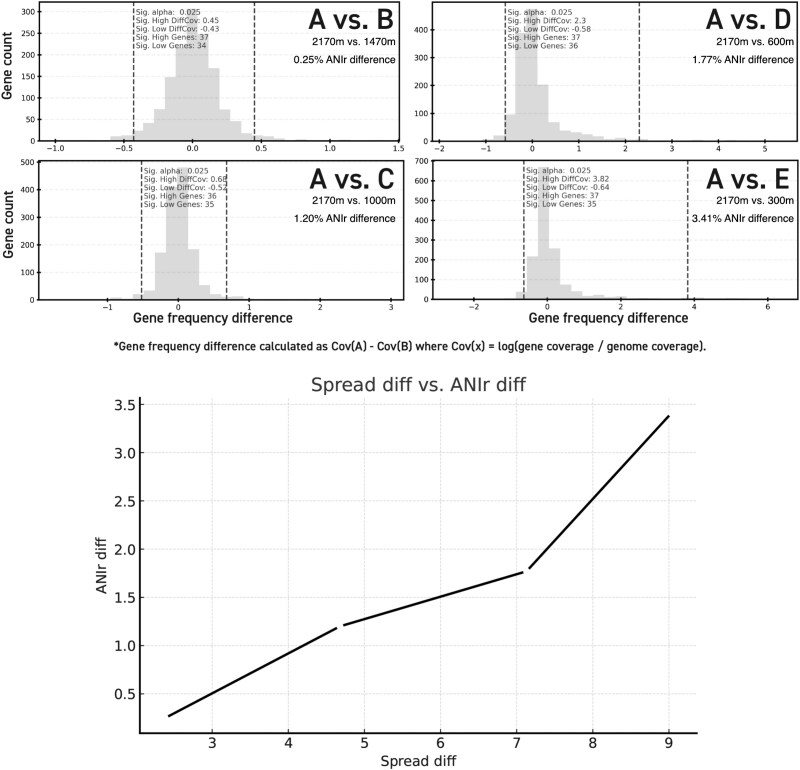
A test for detecting significant gene content differences between samples. Top: Difference in normalized relative gene abundances between the target population detected in the sample shown in Fig. 5A and the target populations detected in the samples shown in Fig. 5B–E. The gene difference distributions between target populations in Sample A vs. Samples B–E for all predicted CDS from the archaeal rMAG discussed in the main text are shown as histogram in light gray. The vertical dashed lines show three standard deviations above and below the mean. Differences were calculated A – B, C, D, or E such that positive gene differences were found at higher frequency in Sample A, and negative gene differences were found at higher frequency in Samples B, C, D, or E. Note that the majority of genes are centered around a difference of 0 indicating, as expected, that the core genes of a species do not change in frequency within separate subpopulations. Bottom: As the ANIr difference between populations increase from A vs. B to A vs. E, the corresponding min. vs. max. values of gene differences also increase in the same order.

To test for gene content differences between populations and samples, the relative gene abundance was normalized by the relative genome abundance in each sample (see Methods for more details). In this way, the relative abundance of the reference genome is used as the reference frame to compare relative gene abundance across samples. This same genome is then held constant as the reference for all sample comparisons to Sample A. Subsequently, the difference in normalized (by the genome abundance) gene abundance between two samples (Sample A and Sample B) was computed for all genes of the same rMAG and used as a probability distribution of the expected difference in gene abundance. That is, the relative abundance of the genome could change between samples (e.g. 10× vs. 20×, in Sample A vs. B, respectively) but the ratio of abundances of the genes should be constant (e.g. 0.5 in our example for A vs. B abundances) unless the gene(s) also changes in frequency within the population or sample (e.g. becomes 30× for increase when the genome abundance is 20×). Assuming that the relative genome abundance follows more closely the core genes of a population than the accessory or transient genes, differences in relative gene abundance normalized in this way largely indicate differences in the frequency of that gene in the population in reference to the core genes of that population. Then, the difference between all genes in the same genome between two samples represents a probability distribution of observing a given difference given all the observed gene differences between two samples (Fig. 8). Consequently, the bottom 2.5% and top 97.5% quantiles from this distribution represent the genes that show the greatest significant change in frequency between two populations (*P* ≤ .05). For this analysis, the normalized relative gene abundance of the archaeal rMAG target populations shown in Fig. 5B–E were subtracted from the relative gene abundance from the target population shown in Fig. 5A such that genes to the left of zero (no difference) were found at a higher frequency in target populations B–E (Sup. Table 1) and genes to the right of zero were found at a higher frequency in target population A (Sup. Table 2; Fig. 8; Sup. File 2). Hypothetical gene functions (“uncharacterized protein,” “NA,” and “No match found”) accounted for 82 of the 147 (55.78%) genes with higher frequency in the target sample population and 89 of the 140 (63.57%) genes with higher frequency in the test sample populations (Sup. File 2, Overview tab). Further, several functions such as peptidases (cellular matter degradation) and mobile elements (e.g. transposases) were more abundant in the deeper populations relative to their shallower counterparts (Sup. Tables 1 and 2), which is consistent with results reported previously [19, 50–53] and further validates the robustness of our approach.

Similarly to the ANIr differences increasing between target populations detected with the same rMAG in different samples, the gene content differences also increased (i.e. note the greater spread in the distributions shown in Fig. 8). With this method, the majority of gene differences were always centered around zero, but the spread of the distribution increased as ANIr decreased. In some instances, a bimodal distribution was observed with a shorter second peak above or below the main peak centered around zero, which could be a signal of two co-existing, but diverging, subpopulations (data not shown) or a cluster of genes present/absent from a sample/environment. As a result, many genes within such a distribution may not reach statistical significance using parametric methods, yet they remain biologically interesting and warrant further investigation. The method presented here, in additional to standard SNV analyses for allele frequency differences, should provide a useful post hoc test for exploring the differences between populations detected in different samples.

## Conclusions

This work introduced the following analytical approaches to identify boundaries between and within populations: (i) identify metagenomic short-read alignments belonging to the target population vs. closely related but nontarget and distinct populations based on a reference/representative sequence of the target population, (ii) generate statistical insights as to when similar target populations identified with the same reference sequence in different metagenomic samples are the same or not, and (iii) normalize via a reference frame approach to identify genes showing significant change in frequency between target populations. A key component of these approaches relies on the ability to automatically identify local minima in the distributions of read mapping results to select percent identity thresholds and thus optimize the selection of short-read alignments belonging to a target population vs. a related but nontarget population. This approach not only enhances the accuracy of population delineation but also provides a robust framework for characterizing intraspecies diversity between metagenomic datasets. The local minima detection presented here could also be further optimized by using, e.g. a sliding window approach to allow for dynamic read filtering thresholds to be utilized across the genome as we suggested previously for single genes [54].

Using these approaches, significant spatial variability among microbial populations across ocean basins and depths was observed, even in cases where the populations differ by <1% in terms of ANIr. These findings suggest that adaptive responses to microscale endemic environmental gradients may influence population dynamics and genetic structure even at relative short geographic distances (vertical and/or horizontal) and similar environments (e.g. similar depths in the oceans). It should be mentioned, however, that when the differences are small such as <0.5% in ANIr, these could just represent the differential abundance of genomovars within species and not necessarily significant sequence divergence. Distinguishing between such scenarios would typically require additional work such as recovery of MAG from each sample and competitive read mapping against all MAGs or employing phylogenetic analysis of long-read sequences and/or SAGs, as we performed recently [13, 14]. The approach outlined here can aid the researcher in identifying interesting samples and species (MAGs) from large datasets for such further detailed analysis. Given also that the genomovar gap is observed around 99.5% for most species (so, ~0.5% ANI difference) and current (rather low) sequencing errors of the common platforms, we would like to suggest that differences in ANIr <0.5% could possibly represent genomovar and strain differential abundance and/or spurious results rather than substantial genomic sequence adaptations (or the latter were initiated only relatively recently), and thus could be given less attention compared to differences of at least 0.5% ANIr. Finally, although bootstrap and permutation tests give, in general, similar results for the same datasets, bootstrap is usually more robust when data are limited (such as when a target species is barely detectable, so the number of mapped reads is relatively low, e.g. below a couple thousands). Thus, we suggest using both tests together and identify interesting species and samples for further analysis when differences with both tests are significant. Additionally, by setting the gene abundance relative to the genome abundance within each sample, we identified specific genes showing significant frequency variations within seemingly similar populations detected in different samples. Therefore, this gene-content approach can provide deeper insights into functional adaptations and genetic diversity dynamics, complementing the ANIr-based analyses to elucidate potential adaptation mechanisms in marine ecosystems. The methodology presented here should be directly applicable to other habitats and similar microbial metagenomic data.

## Supplementary Material

ycag068_Supplemental_Files The Supplementary online material includes additional details on the code developed and the metagenomes used to those mentioned above.

## Data Availability

All data are available in the main text or the supplemental files. The code for computing and plotting the KDE, local minima, and coverage metrics is available from the following GitHub repo: https://github.com/rotheconrad/Metagenomic_Population_Tracking. Zenodo DOI: 10.5281/zenodo.15231818
