# Supplementary material for: An ANIr-based methodology to determine if two sequence-discrete populations are identical and identify cosmopolitan prokaryotic populations
Source: ISME Commun. 2026 Mar 21;6(1):ycag068. doi: 10.1093/ismeco/ycag068 (PMC13098170; doi:10.1093/ismeco/ycag068)
Supplement: ycag068_Supplemental_Files — The Supplementary online material includes additional details on the code developed and the metagenomes used to those mentioned above. [file ycag068_supplemental_files.zip › 07b_GoM-Abundance-supplement_ycag068.pdf]

TITLE: An ANIr-based methodology to determine if two sequence-discrete populations are identical and identify cosmopolitan prokaryotic populations

SHORT TITLE: ANIr-based population distinction

AUTHORS: Roth E. Conrad[1], Luis M Rodriguez-R[2], Blake G. Lindner[3, +], Kenji Gerhardt[4], Konstantinos T. Konstantinidis[1,3,4,*]

[1]Ocean Science & Engineering, School of Biological Sciences, Georgia Institute of Technology, Atlanta, GA, USA.

[2]Department of Chemistry and Biosciences, Aalborg University, Aalborg, Denmark.

[3]School of Civil & Environmental Engineering, Georgia Institute of Technology, Atlanta, GA, USA.

[4]Center for Bioinformatics and Computational Genomics, School of Biological Sciences, Georgia Institute of Technology, Atlanta, GA, USA.

[+]Present address: Department of Chemical Engineering & Applied Chemistry, University of Toronto, Toronto, Ontario, Canada

*Corresponding author:

Konstantinos T. Konstantinidis | kostas.konstantinidis@gatech.edu

School of Civil and Environmental Engineering,

Georgia Institute of Technology,

311 Ferst Drive,

ES&T Building, Room 3321,

Atlanta, Georgia 30332-0512, USA.

Supplemental Figures

Sample	Ocean	ANIr	MNIr	Density	Temp	Salinity	Depth
TAR_ATLx_250m	North Atlantic	94.71	94.29	26.43	18.17	36.56	250
ATL_S0076_0184m	North Atlantic	93.55	93.33	26.44	18.20	36.59	184
GoM_EN54_0150m	Gulf Of Mexico	95.20	95.17	26.61	15.86	36.08	150
ATL_S0078_0301m	North Atlantic	93.88	94.00	26.68	16.06	36.23	301
HOT_xx_0500m	North Pacific	93.67	93.92	26.69	7.06	34.09	500
ATL_S0080_0500m	North Atlantic	94.76	95.17	27.00	12.73	35.72	500
GoM_EN55_0300m	Gulf Of Mexico	95.81	96.00	27.01	11.46	35.41	300
TAR_st142_640m	Gulf Of Mexico	96.08	96.88	27.12	9.76	35.17	640
PAC_GT15_1008m	South Pacific	97.82	98.00	27.14	4.95	34.31	1008
TAR_ATLx_740m	North Atlantic	96.00	96.04	27.15	10.57	35.38	740
HOT_xx_0770m	North Pacific	97.55	98.00	27.15	5.10	34.36	770
TAR_st072_800m	South Atlantic	97.34	98.02	27.29	4.67	34.47	800
GoM_EN56_0600m	Gulf Of Mexico	97.45	97.89	27.33	7.17	34.92	600
HOT_xx_1000m	North Pacific	97.47	98.00	27.38	4.11	34.50	1000
ATL_S0081_1055m	North Atlantic	97.57	98.00	27.54	6.14	35.00	1055
GoM_EN57_1000m	Gulf Of Mexico	98.02	98.66	27.62	4.99	34.93	1000
GoM_EN58_1470m	Gulf Of Mexico	99.47	100.00	27.72	4.28	34.96	1470
GoM_EN59_2170m	Gulf Of Mexico	99.22	100.00	27.73	4.25	34.97	2107

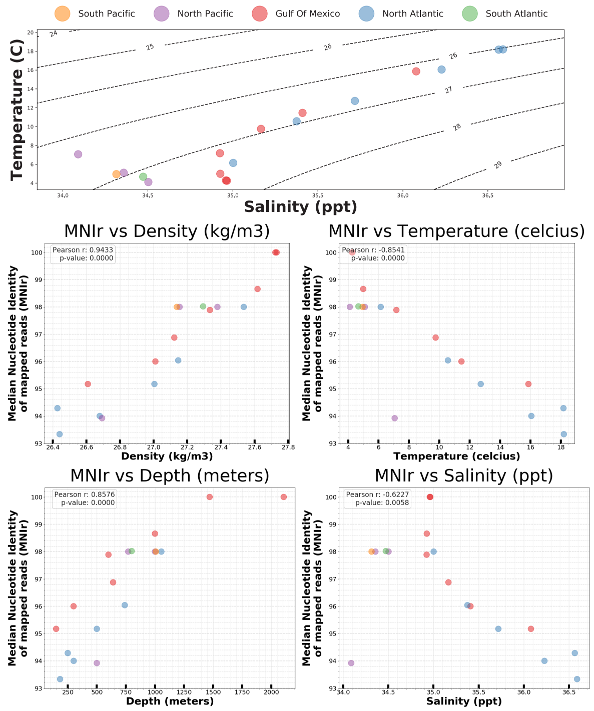

Supplemental Figure 1. Median average nucleotide identity of the target populations in each metagenome sample versus standard sea water geochemical metrics.

Supplemental Tables

Table 1. Predicted functions and their distinct sequence (allele) copy counts in the reference MAG genome for genes with greater normalized relative abundance in the reference population for the comparisons shown in Figure 8. These genes were identified in Figure 8 as those with positive significant differences (right side). The unique gene counts, and functional gene annotations, are from the predicted CDS for the archaeal rMAG discussed in the main text. Each unique CDS that is assigned the same function is counted once for that function and reported in the table. Note this is different than the relative abundance or relative abundance difference that was used to identify these genes. The relative abundance for the comparisons of A vs. B-E shown in Figure 8 could change in two ways. 1) The target population genomes could contain more (or fewer) CDS that received the same functional annotation than the reference population. These could be recent gene duplications or unique copies (paralogs). 2) The increase in abundance of a genomovar carrying variable or genomovar-specific gene content. A fully detailed table of abundance, difference, and additional functional annotation can be found in Supplementary File 2.

Gene Function	Count
uncharacterized protein	34
NA	25
No match found	23
pts system mannitol-specific eiic component	3
oxidoreductase domain-containing protein	3
beta_helix domain-containing protein	3
d-2-hydroxyacid dehydrogenase	3
phosphoenolpyruvate--protein phosphotransferase	2
prd domain-containing protein	2
cchc-type domain-containing protein	2
lrp/asnc family transcriptional regulator	2
abc-f type ribosomal protection protein	2
aldehyde dehydrogenase	2
aaa_16 domain-containing protein	2
glutamine amidotransferase type-2 domain-containing protein	2
bph_2 domain-containing protein	2
pts sugar transporter subunit iia	2
orotidine 5'-phosphate decarboxylase	2
30s ribosomal protein s14	1
phosphomannomutase/phosphoglucomutase	1
ketoacyl-acp synthase iii	1
pyruvate, water dikinase	1
acidppc domain-containing protein	1
mts domain-containing protein	1

atp-synt_c domain-containing protein	1
dna polymerase ii large subunit (dpb1)	1
pkd domain-containing protein	1
peptidase m60 domain-containing protein	1
oligopeptide transport atp-binding protein appd (abc.pe.a)	1
n6_n4_mtase domain-containing protein	1
aminotransferase class i/ii-fold pyridoxal phosphate-dependent enzyme	1
transporter	1
abc transporter atp-binding protein	1
duf4287 domain-containing protein	1
tigr00266 family protein	1
mur_ligase_m domain-containing protein	1
lipid-transfer protein	1
endo3c domain-containing protein	1
thioredoxin domain-containing protein	1
glyco_trans_4-like_n domain-containing protein	1
upf0145 protein cxx70_11340	1
zf-tfiib domain-containing protein	1
pa14 domain-containing protein	1
geranylgeranylglyceryl/heptaprenylglyceryl phosphate synthase	1
n-acetyltransferase domain-containing protein	1
glutamine transport system permease protein glnp	1
mfs domain-containing protein	1
fructose 1-phosphate kinase	1
transposase	1

Table 2. Predicted functions and their distinct sequence (allele) copy counts in the reference MAG genome for genes with greater normalized relative abundance in the target population for the comparisons shown in Figure 8. The table is similar to Table 1 but shows instead the genes with higher abundance in the target rather than the reference population. A fully detailed table of abundance, difference, and additional functional annotation can be found in Sup. File 2.

Higher in Test Samples (Figure 5 B-E)	Count
NA	36
uncharacterized protein	34
No match found	19
phosphomannomutase/phosphoglucomutase	3
pyruvate, water dikinase	3
thioredoxin domain-containing protein	3
dna polymerase ii large subunit (dpb1)	3
50s ribosomal protein l6	2
duf357 domain-containing protein	2
ketoacyl-acp synthase iii	2
5-(carboxyamino)imidazole ribonucleotide mutase	2
geranylgeranylglyceryl/heptaprenylglyceryl phosphate synthase	2
vkc domain-containing protein	2
oligosaccharyl transferase	2
2-aminomuconate deaminase	1
lrp/asnc family transcriptional regulator	1
lipid-transfer protein	1
is5 family transposase	1
epimerase	1
beta_helix domain-containing protein	1
d-2-hydroxyacid dehydrogenase	1
glutamine transport system permease protein glnp	1
3-phosphoglycerate dehydrogenase	1
ef-hand domain-containing protein	1
acidppc domain-containing protein	1
atp-grasp domain-containing protein	1
mastigoneme-like protein	1
oligopeptide transport atp-binding protein appd (abc.pe.a)	1
nadh-quinone oxidoreductase subunit j	1
sufc protein atp-binding protein (sufc)	1
v-type atp synthase subunit f	1
acyl-coa dehydrogenase family protein	1

peptidase m60 domain-containing protein	1
mfs domain-containing protein	1
glutamine-hydrolyzing carbamoyl-phosphate synthase small subunit	1
v-type atp synthase subunit i	1
oligopeptide abc transporter permease (oppc)	1
cupin	1
dna-3-methyladenine glycosylase 2 family protein	1
